# Genome-Wide Analyses of Aspartic Proteases on Potato Genome (*Solanum tuberosum*): Generating New Tools to Improve the Resistance of Plants to Abiotic Stress

**DOI:** 10.3390/plants11040544

**Published:** 2022-02-18

**Authors:** Natalia Sigrid Norero, María Florencia Rey Burusco, Sebastián D’Ippólito, Cecilia Andrea Décima Oneto, Gabriela Alejandra Massa, Martín Alfredo Castellote, Sergio Enrique Feingold, María Gabriela Guevara

**Affiliations:** 1 Laboratory of Agrobiotechnology IPADS (INTA—CONICET), Balcarce B7620, Argentina; norero.natalia@inta.gob.ar (N.S.N.); rey.mariaflorencia@inta.gob.ar (M.F.R.B.); decimaoneto.cecilia@inta.gob.ar (C.A.D.O.); massa.gabriela@inta.gob.ar (G.A.M.); castellote.martin@inta.gob.ar (M.A.C.); feingold.sergio@inta.gob.ar (S.E.F.); 2 Faculty of Agricultural Sciences, University National of Mar del Plata, Balcarce B7620, Argentina; 3 Institute of Biological Research, University of Mar del Plata (IIB-UNMdP), Mar del Plata B7600, Argentina; dippolit@mdp.edu.ar; 4 National Scientific and Technical Research Council, Argentina (CONICET), Buenos Aires C1499, Argentina

**Keywords:** aspartic proteases, phylogeny, intronless, MEME, CIS-elements, abiotic stress

## Abstract

Aspartic proteases are proteolytic enzymes widely distributed in living organisms and viruses. Although they have been extensively studied in many plant species, they are poorly described in potatoes. The present study aimed to identify and characterize *S. tuberosum* aspartic proteases. Gene structure, chromosome and protein domain organization, phylogeny, and subcellular predicted localization were analyzed and integrated with RNAseq data from different tissues, organs, and conditions focused on abiotic stress. Sixty-two aspartic protease genes were retrieved from the potato genome, distributed in 12 chromosomes. A high number of intronless genes and segmental and tandem duplications were detected. Phylogenetic analysis revealed eight *St*AP groups, named from *St*API to *St*APVIII, that were differentiated into typical *(St*API), nucellin-like (*St*APIIIa), and atypical aspartic proteases (*St*APII, *St*APIIIb to *St*APVIII). RNAseq data analyses showed that gene expression was consistent with the presence of cis-acting regulatory elements on *St*AP promoter regions related to water deficit. The study presents the first identification and characterization of 62 aspartic protease genes and proteins on the potato genome and provides the baseline material for functional gene determinations and potato breeding programs, including gene editing mediated by CRISPR.

## 1. Introduction

Aspartic proteases (APs) (EC 3.4.23) are widely distributed among living organisms and viruses [1,2,3,4]. These enzymes have been extensively studied and constitute one of the four superfamilies of proteolytic enzymes. APs are characterized by the presence of two aspartic acid residues located within the conserved Asp-Thr/Ser-Gly motif, responsible for catalytic activity [2,5,6].

The MEROPs database classified APs into 15 families based on their amino acid sequence similarity and grouped them into 5 different clans based on their evolutionary relationship and tertiary structure [7]. Plant APs belong to families A1, A3, A11, and A12 of clan AA and family 22 of clan AD [8]. Most of the plant APs belong to the A1 family, are active at acidic pH, are specifically inhibited by pepstatin A, and present a great structural diversity [8,9]. In silico analysis of the *Arabidopsis thaliana* genome revealed the presence of 51 genes that encode possible APs (*At*AP), 46 of which presented different primary structure characteristics compared to the canonical forms. Based on the sequence of the active sites and the organization of domains, *At*APs are currently sorted into three groups or categories: typical, atypical, and nucellin-like [10].

Typical plant AP precursors present a protein domain of 100 amino acids long known as the plant-specific insert (PSI), which is highly similar to saposin-like proteins [8]. In monomeric typical APs, this domain still remains in the mature enzyme [11], while PSI is removed from the precursors upon activation in most of the heterodimeric typical APs enzymes [8]. Atypical and nucellin-like APs share several common features such as the absence of the internal segment plant-specific insert in their sequence, variable N-terminal regions, differences in active site environment, unusually high cysteine content and localization, and have different primary structure organizations from typical APs [10,12]. Coincidentally with *A. thaliana*, most potential APs sequences from grape and rice genomes correspond to atypical and nucellin-like APs [9]. Regarding typical APs, their expression has been determined simultaneously in several different plant organs, including flowers, leaves, roots, stems, and seeds, as well as in different stages of development [9,13,14,15]. Typical AP functions have been associated with development, growth, lipid metabolism, and protein degradation [16,17].

Only nine atypical and one nucellin-like APs have been partially studied and characterized in *A. thaliana* [17,18,19,20,21,22,23,24,25]. In addition, atypical APs have been reported in other plant species such as *Oryza sativa* [16,26,27,28,29], *Lotus japonicus* [30], *Fagopyrum tataricum* [31,32], *Solanum tuberosum* [33], *Nicotiana tabacum* [34], several carnivorous plants [35,36,37], *Phaseolus vulgaris* [38], and *Vitis vinifera* [39]. Biological functions of atypical APs have been related to plant defense response mechanisms, hybrid sterility, reproductive development, abiotic and biotic stresses, chloroplast homeostasis, and lateral root development [12,13,14,15,16,17]. Current knowledge on the participation of APs in plants’ signaling pathways in response to water deficit and their relationship with plant stress tolerance was summarized recently [40]. These AP roles have been studied in several plant species [21,33,41,42,43,44]. In addition, we have demonstrated that leaves from *A. thaliana* plants overexpressing a typical AP (*At*APA1) showed changes in morphological and physiological features related to water loss deficit [45]. We concluded that *At*APA1 participates as intermediate in the ABA-induced stomatal closure, as well as in the stomatal density regulation conferring tolerance to mild water deficit.

For the last two decades, we focused our research on the biochemical and molecular characterization of *Solanum tuberosum* L. aspartic proteases (*St*APs) [11,46,47,48,49]. Additionally, we reported the role/s of two typical *St*APs (*St*AP1 and *St*AP3) in the plant’s mechanisms in response to water deficit and pathogen attack [45,46,47,48,49]. Potato is an ancestral crop domesticated at least 7000 years ago [50,51], and today it represents the third most important crop for human consumption and is the fourth most important crop in terms of production after wheat, rice, and maize (http://faostat.fao.org/ Accessed date: 25 October 2021). Our laboratory is in the Southeast of the Province of Buenos Aires, Argentina, one of the most important cultivation areas of *S. tuberosum* in this country. Therefore, increasing the knowledge about how potato plants respond to abiotic and biotic stress is crucial for farmers and breeding programs.

The aim of this work was to identify and characterize the AP genes present on the *S. tuberosum* genome, focused on abiotic stress conditions. Data obtained provide new knowledge about gene structure and protein organization, tissue, and subcellular localization of these enzymes in potato plants. In addition, this study provides the baseline material for functional gene analysis and for potato breeding programs, including gene-editing mediated by CRISPR.

## 2. Results and Discussion

### 2.1. StAPs Potato Genome-Wide Identification

The Hidden Markov Model (HMMER3.1) profile, built from 59 aspartic protease amino acid sequences from *A. thaliana* (*At*APs), was tested against the TAIR 10 2010-12-2014 peptide database rendering 78 *At*APs amino acid sequences up to an e-value of 1.5 × 10^−21^ and a score of 77 on full sequence (Table S3). The HMMER 3.1 search allowed the detection of 149 amino acid sequences on the Potato Genome Sequence Consortium Doubled Monoploide 3.4 version (PGSC DM3.4) peptide database with a maximum e-value of 0.0024 and a score of 18.5 on full sequence (Table S4). These corresponded to 97 tentative *St*AP genes.

**Figure 1:**
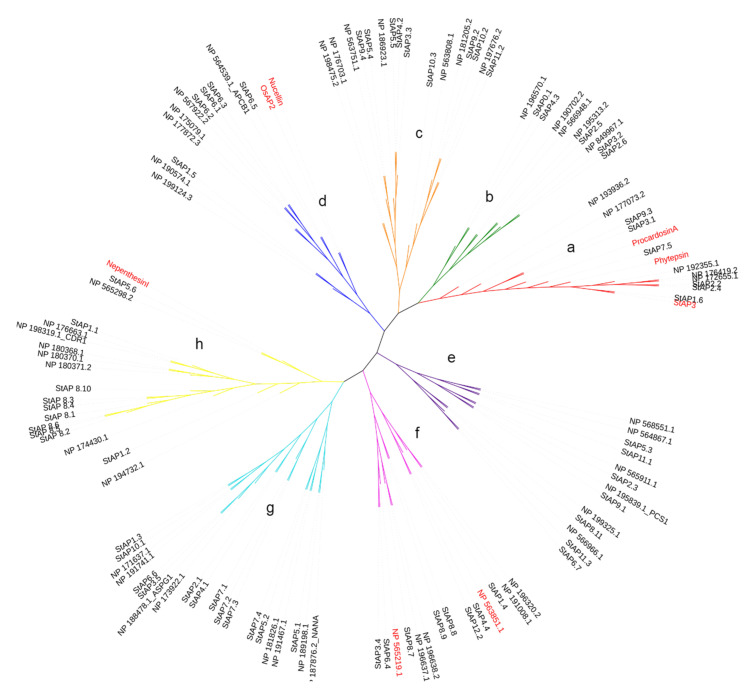
Phylogenetic tree of representative aspartic proteases. The phylogenetic tree was built with a total of 62 and 51 AP aminoacid sequences from *S. tuberosum* and *A. thaliana*, respectively, and 6 reference sequences from *O. sativa* L. (*Os*AP2), *S. tuberosum* L. (*St*AP3), *A. thaliana* L., *C. cardunculos* L. (Cardosin A), *N. gracilis* (Nepenthesin I), and *H. vulgare* L. signed in bold. Cluster names include typical (a), atypical (b, c, e, f, g, and h), and nucellin-like (d) aspartic proteases.

A total of 98 AP amino acid sequences codified by 62 AP genes were retrieved from the potato genome. The longest amino acid sequence was selected from each gene that was confirmed to have the Asp domain (PF00026), including two conserved aspartic acid catalytic residues (Figure 1). From these 149 amino acid sequences, there were 54 sequences with partial or absent Asp domain PF00026 (PFam database) and were thus not taken into consideration for further analyses. The corresponding genes, transcripts and protein names, lengths, and chromosome positions are presented in Table S5.

This AP genome representation is close to what has been previously reported: 51 and 67 *At*APs reported in *A. thaliana* [10,52], 50 APs in *V. vinifera* (*Vv*APs) [9], 67 APs in *P. trichocarp* (*Pt*APs) [53], and 96 APs from *O. sativa* (*Os*APs) [16].

### 2.2. Phylogenetic Analyses

In order to know the phylogenetic relationship between the 62 identified *St*APs and previously reported APs, we constructed a phylogenetic tree with 51 known *At*AP amino acid sequences from the plant model species *A. thaliana* [10] and 6 reference AP amino acid sequences: Nucellin (U87148), Procardosin A (CAB40134), *St*AP3, *Os*AP2 (AAK81699), Nepenthesin I (AB114914), and Phytepsin (CAA39602) (cited in Materials and Methods section).

The phylogenetic analyses evidenced the presence of eight aspartic protease clusters, which were named from “a” to “h” (Figure 1). These clusters grouped typical, nucellin-like, and atypical APs in a similar distribution observed in other plant species [9,16,53]. Cluster “a” represented typical aspartic proteases, with three reference typical aspartic proteases previously cited (*St*AP3, Procardosin A, and Phytepsin) and four *At*APs reported for this category [10]. Cluster “a” included six *St*APs: *St*AP1.6, *St*AP7.5, *St*AP2.2, *St*AP2.4, *St*AP3.1, and *St*AP9.3. Similar numbers of typical APs are present in grape (*V. vinifera*) [39], poplar (*Populus* sp.) [53], and rice (*O. sativa*) [16]. As expected, *St*AP1.6 clustered closely with its homolog from *S.tuberosum* ssp. *tuberosum* cv. *Pampeana*, *St*AP3 [11]. *St*AP2.2 and *St*AP 2.4 were related with two typical *At*APs, NP_176419, a seed-specific AP [54], and NP_172655, while *St*AP7.5 was closely related with Phytepsin from barley (*Hordeum vulgare*) [55] and Procardosin A from cardon (*C. carduculus* L.) [56].

Cluster “d” represents nucellin-like APs, since it included the two reference proteins Nucellin (U87148) from barley [57] and *Os*AP2 (AAK81699) from rice [27] and four AP proteins from *A. thaliana* [10]) for this category. This cluster grouped four *St*APs: *St*AP6.1, *St*AP6.2, *St*AP6.3, and *St*AP6.5. Possible orthologous pairs present in this cluster were *St*AP6.2/NP_567922.2 without known function and *St*AP6.5/NP_564539.1_APCB1 involved in triggering autophagy and defense resistance [58]. Clusters “b”, “c”, “e”, “f”, “g”, and “h” grouped atypical aspartic proteases. Atypical aspartic proteases are divided into six categories [10] based on structural primary sequence features that could be reflected in the clustering.

Cluster “b” and “c” included mostly *At*AP proteases (8 out of 10) from atypical category C4 (with C-terminal extension), 2 *At*APs from category Cx (not assigned type), and 1 from category C1. Cluster “b” included *St*AP3.2 and *St*AP2.6 related to NP_849967.1, *St*AP2.5 joined with NP_195313.2, all from category C4, which present a similar primary structure with a C-terminal extension, *St*AP4.3 (also with a C-terminal extension), and *St*AP0.1, closely related to NP_196570.1 (Category Cx). Cluster “c” presented *St*APs related to *At*AP category C4, such as *St*AP11.2/NP197676.2, *St*AP10.3/NP_563808.1, and *St*AP3.3/*St*AP4.2/*St*AP5.5/NP186923.1, while *St*AP10.2 and *St*AP9.2 grouped with NP181205.2 (category C1). All these last three sequences present a C-terminal extension characteristic of Category C4.

Cluster “e” included three *At*APs from category C1 related with *St*AP5.3, *St*AP6.7, *St*AP11.1, *St*AP11.3, and three *At*APs from category C2 (in [10] with a serine-rich pro sequence). These *At*APs were close related to a *St*AP each NP_565911.1/*St*AP2.3, NP_195839.1_PCS1/*St*A9.1, and NP19325.1/*St*AP8.11, however, none of the *St*APs presented the characteristic serine pro sequence.

Cluster “f” included all three *At*APs from category C5 (“atypical without the DTS active site motif”), which were related with *St*AP1.4, *St*AP4.4, and *St*AP12.1; two *At*APs from category C3, which includes NP196638.2 homolog to CND41 [34], were closely related with *St*AP8.7, *St*AP8.8, and *St*AP8.9, and NP_565219.1 from category Cx was related with *St*AP6.4 and *St*AP3.4.

Cluster “g” grouped *At*APs from category C3 (known as “atypical with long prosegment”) and two sequences from category Cx and C1. This cluster contained NP_188478.1 ASPG1, which participates in drought tolerance and protein degradation [21], related with *St*AP3.5 and *St*AP6.6. In this cluster was also present NP_187876.2_NANA, closely related with *St*AP5.1, which plays regulatory functions in chloroplasts [22].

Cluster “h” includes *At*APs mostly from category C1, two *At*APs from category Cx, and one *At*AP from category C2. Among category C1 there were present NP_198319.1_CDR1 and NP_176663.1, closely related to *St*AP1.1. NP_198319.1_CDR1 is a rice ortholog linked to disease resistance mechanisms in *A. thaliana* [26]. Cluster “h” also grouped Nepenthesin I from *N. distillatoria* [59] reference sequence closely related to *St*AP5.6.

**Figure 2:**
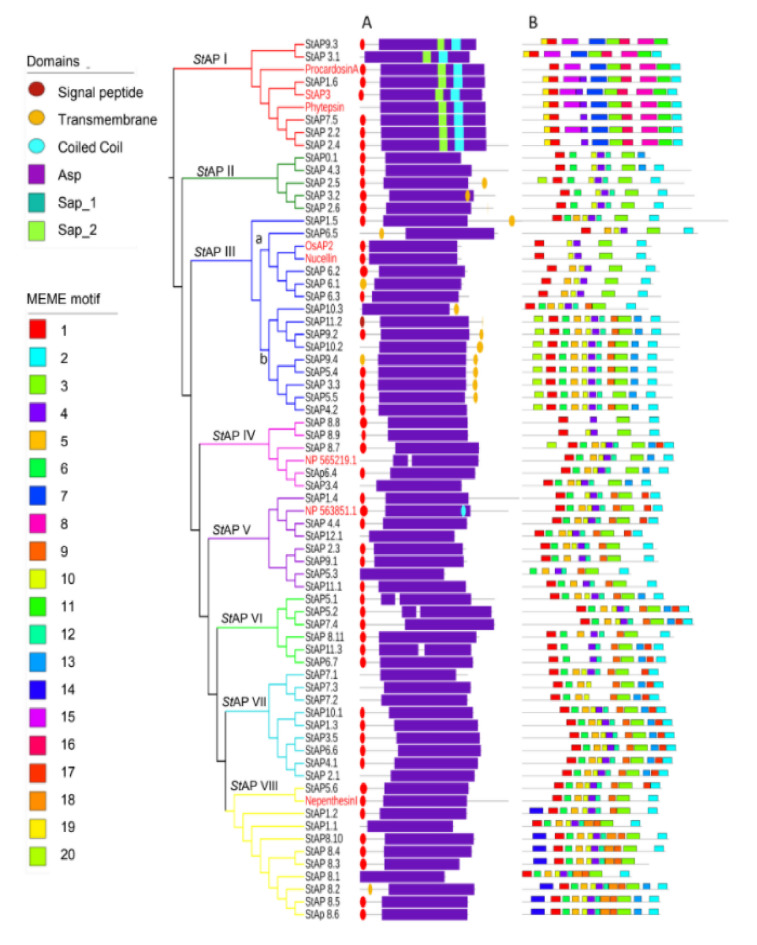
Phylogenetic tree of 62 representative aspartic proteases from *S. tuberosum*. The tree includes 8 reference aspartic proteases from *O. sativa* L. (*Os*AP2)*, S. tuberosum L*. (*St*AP3), *A. thaliana* L. (NP_565219.1 and NP_563851.1)*, C. cardunculos*. (Procardosin A), *N. gracilis* (NepenthesinI), and *H. vulgare* (Phytepsin) indicated in red. Group names and branches are indicated with different colors. (**A**) Pfam Domains; (**B**) MEME motifs (specified in Table S6).

To analyze *St*APs phylogenetic relation together with protein and gene structures, an additional phylogenetic tree was built among the identified *St*APs and eight reference APs from *A. thaliana*, *H. vulgare*, *N. distillatoria*, *O. sativa*, and *C. cardunculus* (Figure 2). *St*APs were grouped in eight clusters, named from *St*API to *St*APVIII, with a similar distribution to Figure 1. *St*API grouped sequences as described in cluster “a” (Figure 1) (*St*AP1.6, *St*AP7.5, *St*AP2.4, *St*AP2.2, *St*AP9.3, and *St*AP3.1). *St*APIII presented two subgroups, one that was named *St*APIIIa, which included nucellin-like *St*APs (*St*AP6.5, *St*AP6.1, *St*AP6.2, and *St*AP6.3) as in cluster “d” (Figure 1), and another subgroup, *St*APIIIb, representing atypical sequences from cluster “c” (*St*AP10.2, *St*AP9.2, *St*AP11.2, *St*AP10.3, *St*AP5.4, *St*AP9.4, *St*AP3.3, *St*AP4.2, *St*AP5.5). In both phylogenetic trees, some atypical sequences related to categories C4 and Cx were observed (Cluster “b” and “c” from Figure 1 and Cluster *St*APII and subgroup *St*APIIIb from Figure 2). A similar *At*AP distribution was obtained in a phylogenetic tree where *Populus* and grape AP amino acid sequences were studied (Figure 1 in [53]).

To analyze *St*APs phylogenetic relation together with protein and gene structures, an additional phylogenetic tree was built among the identified *St*APs and eight reference APs from *A. thaliana*, H., *N. distillatoria*, *O. sativa*, and *C. cardunculus (*Figure 2). *St*APs were grouped in eight clusters, named from *St*API to *St*APVIII, with a similar distribution to Figure 1. *St*API grouped sequences as described in cluster “a” (Figure 1) (*St*AP1.6, *St*AP7.5, *St*AP2.4, *St*AP2.2, *St*AP9.3, and *St*AP3.1). *St*APIII presented two subgroups, one that was named *St*APIIIa, which included nucellin-like *St*APs (*St*AP6.5, *St*AP6.1, *St*AP6.2, and *St*AP6.3) as in cluster “d” (Figure 1), and another subgroup, *St*APIIIb, representing atypical sequences from cluster “c” (*St*AP10.2, *St*AP9.2, *St*AP11.2, *St*AP10.3, *St*AP5.4, *St*AP9.4, *St*AP3.3, *St*AP4.2, *St*AP5.5). In both phylogenetic trees, some atypical sequences related to categories C4 and Cx were observed (Cluster “b” and “c” from Figure 1 and Cluster *St*APII and subgroup *St*APIIIb from Figure 2). A similar *At*AP distribution was obtained in a phylogenetic tree where *Populus* and grape AP amino acid sequences were studied (Figure 1 in [53]).

To analyze *St*APs phylogenetic relation together with protein and gene structures, an additional phylogenetic tree was built among the identified *St*APs and eight reference APs from *A. thaliana*, H., *N. distillatoria*, *O. sativa*, and *C. cardunculus (*Figure 2). *St*APs were grouped in eight clusters, named from *St*API to *St*APVIII, with a similar distribution to Figure 1. *St*API grouped sequences as described in cluster “a” (Figure 1) (*St*AP1.6, *St*AP7.5, *St*AP2.4, *St*AP2.2, *St*AP9.3, and *St*AP3.1). *St*APIII presented two subgroups, one that was named *St*APIIIa, which included nucellin-like *St*APs (*St*AP6.5, *St*AP6.1, *St*AP6.2, and *St*AP6.3) as in cluster “d” (Figure 1), and another subgroup, *St*APIIIb, representing atypical sequences from cluster “c” (*St*AP10.2, *St*AP9.2, *St*AP11.2, *St*AP10.3, *St*AP5.4, *St*AP9.4, *St*AP3.3, *St*AP4.2, *St*AP5.5). In both phylogenetic trees, some atypical sequences related to categories C4 and Cx were observed (Cluster “b” and “c” from Figure 1 and Cluster *St*APII and subgroup *St*APIIIb from Figure 2). A similar *At*AP distribution was obtained in a phylogenetic tree where *Populus* and grape AP amino acid sequences were studied (Figure 1 in [53]).

The remaining clusters correspond to the rest of atypical APs: *St*APIV includes five *St*APs and the reference NP_565219.1 aspartic protease; *St*APV includes seven *St*APs and the reference NP_563851.1_AED3 aspartic protease; *St*APVI includes six *St*APs; *St*APVII includes nine *St*APs. Finally, *St*APVIII includes 10 *St*APs and the Nepenthensin I reference AP.

### 2.3. StAPs Domains and Conserved Motifs

In order to characterize *St*APs at the primary structure level, the presence of domains and conserved motifs were analyzed on the 62 amino acid sequences (Figure 2A, B).

The main *St*APs domains scanned with the Pfam database are shown in Figure 2A, while Figure 2B provides complementary primary sequence-structure information with motifs assessed with Multiple EM for Motif Elicitation (MEME) online software. The majority of *St*APs presented a signal peptide and a pro sequence segment, in accordance with previous reports for typical, atypical, and nucellin-like aspartic proteases, [9,10,12,13,53,60]. Transmembrane signals at the C-terminal end were presented exclusively in Aps from *St*APII and *St*APIII groups. Figure 2A also showed aspartic domains (Asp) (PF00026) that contained the catalytic aspartate at the active site in all *St*APs. Xylanase inhibitor domains (PF14543), called TAXI_N and TAXI_C [61,62,63], were found overlapping with the Asp domain, and they were thus not included in Figure 2A. Both TAXI domains were present in all *St*APs except for the TAXI_C domain that was absent in typical *St*APs. These domains were described as jointly necessary for creating a catalytic pocket for xylanase cleavage, a product generated by pathogens that destroys plant cells [62,63,64]. The role of these domains in aspartic proteases require further studies [64]. Another characteristic feature is the presence of the PSI domain or Saposin-like SapB 1 and SapB 2 domains [65] in all aspartic protease sequences from group *St*API, coincident with the reported for the close related typical AP reference sequences.

Regarding *St*AP motifs assessed with MEME online software (Figure 2B), the number of motifs per protein ranged from 4 to 12, with a length between 15 and 50 amino acids long.

A total of six *St*AP sequences clustered in Group *St*API along with the typical aspartic proteases, presented at least six group exclusive motifs, such as 7, 8, 11, 15, 16, and 19. Motifs 8 and 16 were detected as part of PSI domains SapB_1 and SapB_2 respectively. While motifs 1, 8, 11, and 16 were characterized by the presence of conserved cysteine residues.

Subgroup *St*APIIIb exhibited motif 20 at the N-terminal region in most sequences, and a remarkable characteristic is the substitution of hydrophobic by polar amino acid (glutamine) at the surroundings of the first catalytic site (Table S1).

Group *St*AVIII related to NepenthensinI presented 2 distinctive conserved motifs, motif 14 at the N-terminal region and motif 18, both of which were present in 7 out of 10 *St*APVIII sequences (Figure 2B).

Additionally, some motifs were shared by atypical and nucellin-like AP groups (Groups from *St*APII to *St*APVIII) but were absent in Typical *St*API group, such as motifs 6, 10, 12, and 13. Interestingly, motif 10 presented a segment that has been previously reported to be an important conserved sequence GCGYDQ in nucellin-like APs [10], but although the motif showed highly conserved Gly and Cys residues in the majority of *St*APs, this six amino acid sequence was present in only two sequences of the nucellin-like, *St*AP6.1 and *St*AP6.2 (in *St*APIIIb).

Motif 5 was present in *St*APs from the majority of groups. Sequence inspection allowed the identification of a conserved Tyr residue of the flap region (Tyr75 of pepsin numbering [10]) in all 62 *St*AP sequences, although the motif was not present in groups *St*API and *St*APII. It has been previously reported that nucellin-like APs had a conserved sequence QCDYE in the vicinity of Y75 [10]. Coincidentally, this sequence was found exclusively in *St*APs that clustered with the reference nucellin-like APs in group *St*APIIIa.

Finally, some motifs such as 1, 2, 3, and 4 were present in all eight *St*APs groups, highlighting the importance of these conserved overall regions in aspartic proteases, while a deeper analysis showed group or subgroup distinctive amino acid sequences. Motif 2 was present at the C-terminal region of most 62 *St*APs. A closer inspection of motif 2 confirmed the presence of HTVFD amino acid sequence exclusively in five out of six *St*APs from Group *St*API. This short amino acid sequence was reported previously to be exclusive of typical *St*APs [10]. Motif 4 comprises the GLIGL sequence reported to be in the loop that forms the active site [3,10].

Motif 1 and 3 contained the DT/SG from the first and second catalytic sites, respectively, and were both present in all 62 *St*APs, except *St*AP5.3, which, although lacking motif 1, presented the DTG triad of the catalytic site.

The surroundings of the active sites of *St*APs were distinctive and displayed the three characteristic variations reported previously for typical, nucellin-like, and atypical APs.

The main sequence features surrounding the characteristic first and second catalytic aspartate sequences and the C-terminal end that characterize typical, nucellin-like, and atypical aspartic proteases in potato were detected (as indicated in Table S1). The conserved amino acid signature assigned to typical aspartic proteases was found in *St*API at the first catalytic aspartate (hydrophobic-hydrophobic-hydrophobic (F)-DTG-serine-serine) and in the C-terminal region, which includes the characteristic saposin-like sequence HTVFD (except for *St*AP9.3). Most typical aspartic proteases presented a DSG sequence in the second catalytic aspartate, which was not present in *St*AP9.3 and *St*AP3.1, which had DTG instead. Moreover, the C-terminal end of amino acid sequences in this group confirmed the absence of a Cys residue, which was observed in most sequences of the rest of the groups.

*St*APs from cluster “d” and subgroup from cluster *St*APIII presented a similar first catalytic aspartate signature (DϕDTGS(D/N/T)) tha was reported in other species for nucellin-like aspartic proteases (DϕDTGS(D/E)) [10,53] (Table S3). While *St*APs from *St*APII, *St*APIIIb, and *St*APIV to *St*APVIII presented the reported first catalytic aspartate signature for atypical APs (ϕϕDTGS(D/E) [10,12,53], 15 out of 51 atypical *St*APs sequences were more diverse (ϕ(ϕ/A)D(T/A/S)GS(D/S/N/E/Q)), which could probably be reflected in their diversity of functions.

### 2.4. Cysteine Distribution and Glycosylation Sites

Regarding the presence of cysteines in *St*APs, the cysteine content was similar within AP groups, as previously reported [10,12]. It was found that 13 cysteines were the most frequent number, with an upper and lower limit of 9 to 17.

Mature typical APs present six conserved Cys residues involved in three disulfide bonds that are crucial to stabilizing the three-dimensional structure of the enzymes [66]. The plant-specific insert (PSI) also has six cys residues that form three disulfide bridges with the main role of conferring rigidity for antimicrobial activity [65]. In agreement, conserved Cys residues were present in all sequences from group *St*API. Most of the group *St*API conserved cysteine residues were present within motifs 1, 8, 11, and 16. Cys residues from motifs 8 and 16 belong to the PSI sequence.

The Cys-rich region located between the first catalytic site and the conserved Tyr75 (of pepsin numbering, [10]) described in atypical and nucellin-like APs was coincident with motifs 5 and 6. Motif 5 was present in *St*APs from all groups except *St*API and *St*APII and exhibits a conserved Cys residue, while motif 6 is present in all groups except in *St*API and has two conserved Cys of the Cys rich region.

Conserved Cys residues described for atypical and nucellin-like AP were also found in motifs 10, 12, and 13. Motif 17 in groups *St*APIV to *St*APVIII included a conserved Cys that was present in all 62 *St*APs. Although the structures of both typical and nucellin-like proteins have not yet been determined experimentally, molecular modeling predictions for a Nepethesin I protein indicate that the number of disulfide bonds might be similar to those of typical APs [35].

N-linked glycosylation plays an important role in AP biological functions [67]. Typical three-dimensional AP structures have been solved and present two or even three conserved glycosylation sites [66]. *St*API N-linked glycosylation sites were present within the conserved motifs 7, 11, and 15. It was reported that the PSI of populus APs presents a glycosylation site, which was also predicted on *St*AP in motif 8 [53].

Although the structures of atypical and nucellin-like APs are yet to be available, the presence of a glycosylation site has been determined [53]. Interestingly, opposite to what is observed for the presence of glycosylation sites in typical APs within conserved motifs, an atypical AP glycosylation site was found to be in a non-motif region right before motif 10.

### 2.5. StAPs Cellular and Subcellular Predicted Location

Primary protein structure was employed to predict cellular and subcellular location of *St*APs. Most *St*APs were predicted to be localized on vacuole (29 *St*APs), plasma membrane (27 *St*APs), extracellular space (32 *St*APs), Golgi apparatus (21 *St*APs), and cytoplasm (14 *St*APs), while a few were located in the nucleus (9 *St*APs), chloroplast (3 *St*APs), peroxisome (2 *St*APs), and mitochondria (1 *St*APs) (Table S7). About 85% of the 62 *St*APs were predicted to be multi-located. *St*APs’ multilocation could be reflecting their possibility to act in different tissues and/or stress conditions. One of the functions of aspartic proteases is to process enzymes and includes turnover and enzyme activation [68].

Aspartic proteases from *St*API were preferentially predicted to be located on vacuole as other typical APs reported in barley [69], castor bean (*Ricinus communis*) [70], and *A. thaliana* [71]. To a lesser extent, typical APs were also predicted to be in the extracellular space coincident with those reported for tomato (*Solanum lycopersicum*) [72] and tobacco (*Nicotiana tabacum*) [73]. Finally, nucellin-like (Cluster *St*APIIIa) and atypical aspartic proteases (Clusters *St*APIIIb and *St*APIV to *St*APVIII) had been predicted to be in several cellular and subcellular locations, especially the plasma membrane, extracellular space, and the Golgi apparatus. Several examples of atypical and nucellin-like have widespread distribution in other plant species [17].

### 2.6. Chromosome Location

**Figure 3:**
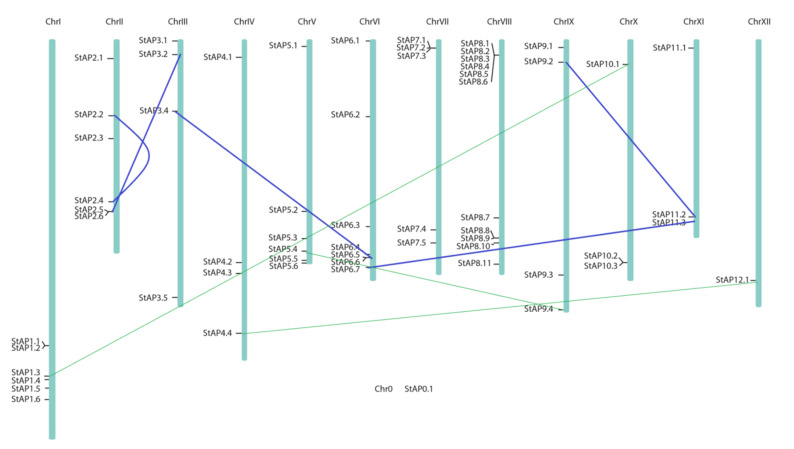
Chromosome distribution of 62 *St*AP genes. Potato physical map (in bp) built by Agrobiotech tools based on PGSC DM3.4 sequence [50]. Blue lines connect genes in segmental duplications blocks previously reported [80], and green fine lines represent possible cases of segmental duplication detected.

*St*APs were distributed along all 12 potato chromosomes, the majority of them localized in chromosomes I, II, V, VI, VII, and VIII (Figure 3). Some AP genes were grouped closely, and others were located in tandem arrays as it could be observed with *St*AP1.1 and *St*AP1.2; *St*AP2.5 and *St*AP2.6; *St*AP6.5 and *St*AP 6.6; *St*AP 7.1 to *St*AP 7.3; *St*AP 8.1 to *St*AP 8.6; *St*AP 8.8, and *St*AP 8.9. However, the *St*AP0.1 chromosome location could not be determined on *S. tuberosum* group Phureja DM1-3 516 R44 genome (version 4.3 [74]). In order to find its probable location, a blast search on the NCBI database was performed. The results obtained showed that this sequence matched a sequence on chromosome 4 of *S. tuberosum* cultivar Solyntus (sequence ID: CP055237.1, Id: 4000/4130(97%); gaps: 32/4130) and on the same chromosome in *S. pinnelli*, and *in S. lycopersicum*.

Segmental and tandem duplications have played a fundamental role in gene family expansions and functional diversification [75]. Wang et al. (2018) investigated the variation in gene family sizes across species and the likely factors contributing to the variation, using the Solanaceae family as a model and Pfam domain families as a proxy for gene families [76]. They found that genes in high- and low-variability gene families tend to be duplicated by tandem and whole genome duplication, respectively. This could be the case of *St*AP genes, where gene duplications and tandem arrays were found (Figure 3, Table S2). Tandem array distribution has also been recently described in APs from *A. thaliana* [77], *V. Vinifera* [9], and *P. trichocarpa* [53].

As observed in the phylogenetic tree (Figure 2), clusters grouped highly similar proteins codified by genes located in different chromosomes. In some cases, it could be attributed to segmental duplications (blue lines in Figure 3), as it was confirmed on the potato genome duplication database (Table S2). In all these cases, a negative selection or purifying selection (Ka/Ks<1) was found with a Ka/Ks 0.211 to 0.24. We also tested other potential gene segmental duplications by using the criteria of a) the length of the aligned sequence covers (at least 75% of the longer gene) and b) a similarity of aligned regions equal or superior to 75% [78]. We found potential cases of segmental duplications for some pairs of genes: *St*AP5.4 and *St*AP9.4; *St*AP12.1 and *St*AP4.4; *St*AP1.3 and *St*AP10.1 (green lines in Figure 3), which presented a Ka/Ks<1 from 0.14 to 0.18 and approximately a duplication date of 24.9 MYA [79].

Segmental and tandem duplications observed in *St*APs genes (approximately 19 of 62) were lower than what was reported in AP genes from *P. trichocarpa* (52 out of 62), however, both segmental duplication events were under purifying selection force. It had been proposed that this could mean the retention of their ancestral functions and redundancy, which will also be related to the sequence environment where they were located [16,53]. Thus, the sequence environment plays an important role and could lead to changes in gene expression patterns of the duplicated copies, as could be the cis-acting regulatory elements that would be analyzed in the next section.

### 2.7. StAP Gene Structure

**Figure 4:**
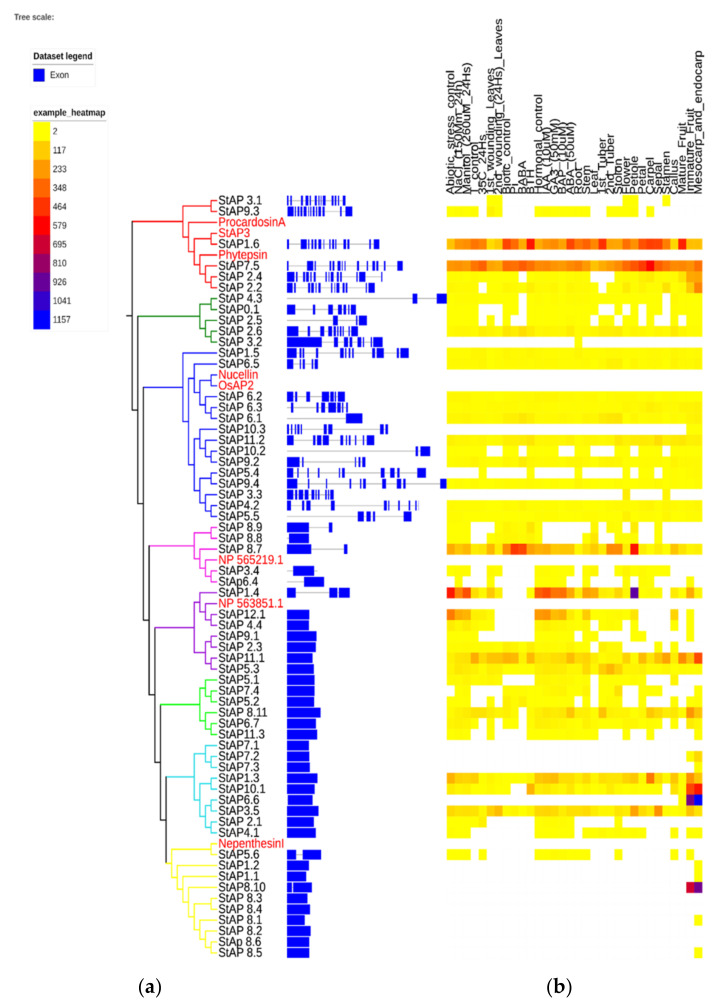
Aspartic proteases exonic and intronic structure and gene expression heatmap. The phylogenetic tree was built with 62 *St*AP amino acid sequences. (**a**) Exon and intron structures were built based on the predicted primary longest transcript structure, and (**b**) RNA-Seq data from different libraries from double monoploid *S. tuberosum* group Phureja DM1-3 516 R44 clone (DM) were indicated as “number of fragments per kilobase of exon per million fragments mapped” (FPKM) by color scale [50]. The phylogram was built with the iTol program [81,82]. Names of genes are colored based on the group membership in Figure 1.

Structural gene analyses were performed comparing exon–intron organization based on the predicted longest primary transcript structure of the 62 *St*AP nucleotide sequences. Twenty-seven intronless *St*AP genes were found, representing almost 44% of the total *St*AP genes (Figure 4). These were mostly grouped in clusters related to atypical APs, such as *St*APV, *St*APVI, *St*APVII, and *St*APVIII. The other four clusters contained *St*AP genes with numerous introns (up to 13), included mainly in *St*API (related with typical Aps), *St*APII (atypical), and *St*APIIIa and *St*APIIIb (related with nucellin-like and atypical Aps, respectively). A similar intron–exon distribution among categories has been reported in rice [16], populus [53], and *A. thaliana* [77].

### 2.8. StAP Expression Analyses

In the last two decades, different studies have reported the participation of APs in plants exposed to adverse environmental conditions such as heat [83,84], osmotic and salt stresses [85], wounding [86], UV-B [13,87,88], and drought [13,21,42,45].

The expression analyses of 62 *St*APs were retrieved from the PGSC DM1.3 RNASeq database to characterize *St*APs and to identify candidate genes that could be involved in different abiotic stresses.

Twenty-six out of sixty-two genes presented a constitutive expression in different tissues or organs (Figure 4). Some *St*AP genes revealed preferential expression and tissue-specific patterns. High constitutive expression levels in most plant organs and tissues were associated with clusters *St*API (with *St*AP1.6, *St*AP7.5), *St*APIV (with *St*AP8.7), *St*APV (with *St*AP1.4, *St*AP7.5, *St*AP11.1), and *St*APVII (*St*AP1.3 and *St*AP3.5), while the highest transcript level was related to cluster *St*APVI (*St*AP1.4, *St*AP11.1) on petiole, to cluster *St*APVIII (with *St*AP8.10) on fruits, and to cluster *St*APVII (with *St*AP10.1 and *St*AP6.6) on mesocarp and endocarp tissues. 

There were five genes without expression in any of the reported conditions or tissues (*St*AP7.1, *St*AP8.2, *St*AP8.3, *St*AP8.4, *St*AP8.6), which could mean that they might be induced in other conditions such as other plant physiological stages or stress sources. Alternatively, the lack of expression might suggest that they could in fact be pseudogenes. Interestingly, four of these genes were grouped in *St*APVIII with the other five genes that were expressed in mesocarp, endocarp, and/or fruit, which points out a tight regulation.

An exception to the above-mentioned behavior of group *St*APVIII was *St*AP5.6 (homolog to Nepenthesin I), which showed expression in several tissues including root, stem, callus, immature fruit, and upregulation after abiotic stress (including salt, mannitol, and temperature treatments) and under hormonal treatment mainly by Gibberellic acid (GA3) and 6-Benzylaminopurine (BAP). This broad expression profile could be related to a role as processing and/or degradative enzyme [8].

Approximately 21% of *St*APs modified their gene expression profile under salt, osmotic, or temperature treatments. It was observed that those *St*AP genes with increased expression after NaCl and mannitol treatments also presented increased transcription profiles after GA3, BAP, and Abscisic Acid (ABA) treatments. The induced genes included *St*AP1.6, which showed the highest induction of transcript levels under NaCl and mannitol treatments, together with *St*AP11.1, *St*AP5.3, and *St*AP8.11. On the other hand, *St*AP1.4, *St*AP12.1, *St*AP1.3, *St*AP8.7, *St*AP4.4, and *St*AP10.1 were repressed.

Regarding heat treatments, 13 genes presented changes in their expression profile. Most of these genes were repressed except for *St*AP7.5, *St*AP2.6, *St*AP2.1, and *St*AP8.7, which exhibited different degrees of induction. Interestingly, some AP genes, orthologous to the ones described in this work, were expressed in similar environmental stress conditions. In a transcriptomic study, it was reported that an *At*AP gene *At*1g62290 (a possible ortholog to *St*Ap7.5) was induced in plants overexpressing the heat shock transcription factor HSfA2 [89]. Interestingly, HSfA2 overexpressing plants were tolerant to high temperatures, as well as to both salt and osmotic stress conditions. The authors did not analyze the role of these AP genes, but they concluded that genes that resulted regulated by HSfA2 could be implicated in multiple stress tolerance responses. Expression analysis of the AP gene superfamily in grape (*V. vinifera*.) identified a total of 19 *Vv*APs that responded to at least one abiotic stress condition. Specifically, the *VvAP21* gene was upregulated in plants treated with NaCl 250 mM during 24 h, in accordance with its possible ortholog, *St*AP1.6 [9]. In another work, the same group overexpressed *VvAP17*, obtaining enhanced salt and drought stress-tolerant plants [85]. In detail, overexpressed *VvAP17* lines exhibited an incremented level of the ABA hormone, a reduced stomatal aperture size, longer primary roots, as well as higher activities of several antioxidant enzymes such as catalase, superoxide dismutase, and peroxidase. A possible ortholog to *VvAP17* is *St*AP4.3, which also showed upregulation after salt and mannitol treatment. These results suggest that *St*APs hormonal expression profiles are related to abiotic stress.

The double monoploid reference genome DM1-3516-R44 is susceptible to *P. infestans* [50]. The analyses of the biotic stress expression profile revealed at least 16 genes that were affected by *P. infestans* treatment. Most of them were downregulated and included *St*AP1.6, *St*AP7.5, *St*AP2.2 (from *St*API), *St*AP8.11, *St*AP5.2 (from *St*APVI), *St*AP2.6 (from *St*APII), *St*AP11.1, *St*AP2.3 (from *St*APV), *St*AP9.2 (from *St*APIIIb), and *St*AP8.9 (from *St*APIV). Typical aspartic proteases have been implicated in plant defense against pathogens. Thus, *St*AP3 protein (homolog to *St*AP1.6) accumulates in intercellular fluids after pathogen infection, and its levels are higher in a field-tolerant cultivar (cv. Pampeana) compared to a susceptible cultivar (cv. Bintje). It has also been shown that *St*AP3 inhibits the germination of *P. infestans* and *F. solani* spores in in vitro experiments [11,48]. The PSI domain *St*AP and procardosin A has been responsible for membrane interaction and permeabilization at low pH [90,91] and in alipid-composition-dependent manner [65,91].

Five genes grouped in cluster *St*APIII had a slight expression increase after *P. infestans* infection (*St*AP11.2, *St*AP6.1, *St*AP6.2, *St*AP6.3, *St*AP3.5), and only *St*AP8.7 (*St*APIV) showed strong upregulation. Some of these genes, *St*AP8.7 and *St*AP6.1, were also upregulated after BABA treatment, related to pathogen defense induction [92]. While *St*AP2.6, *St*AP9.2, and *St*AP7.5 presented an increase in their expression profile under BABA treatment, an opposite effect was observed with *P. infestans* infection and BTH treatment (a salicylic acid analog with SAR response signaling) [93].

Regarding BTH treatment, we observed upregulation in *St*AP1.6 and *St*AP2.2 (cluster *St*API), *St*AP10.1, *St*AP1.3 (cluster *St*APVII), *St*AP11.2 (cluster *St*APIII), and *St*AP8.9 (cluster *St*APIV), while downregulation was observed in at least six genes (*St*AP8.11, *St*AP5.2, *St*AP2.6, *St*AP7.5, and *St*AP8.7) for this treatment (Figure 4).

### 2.9. Cis-Acting Regulatory Elements

**Figure 5:**
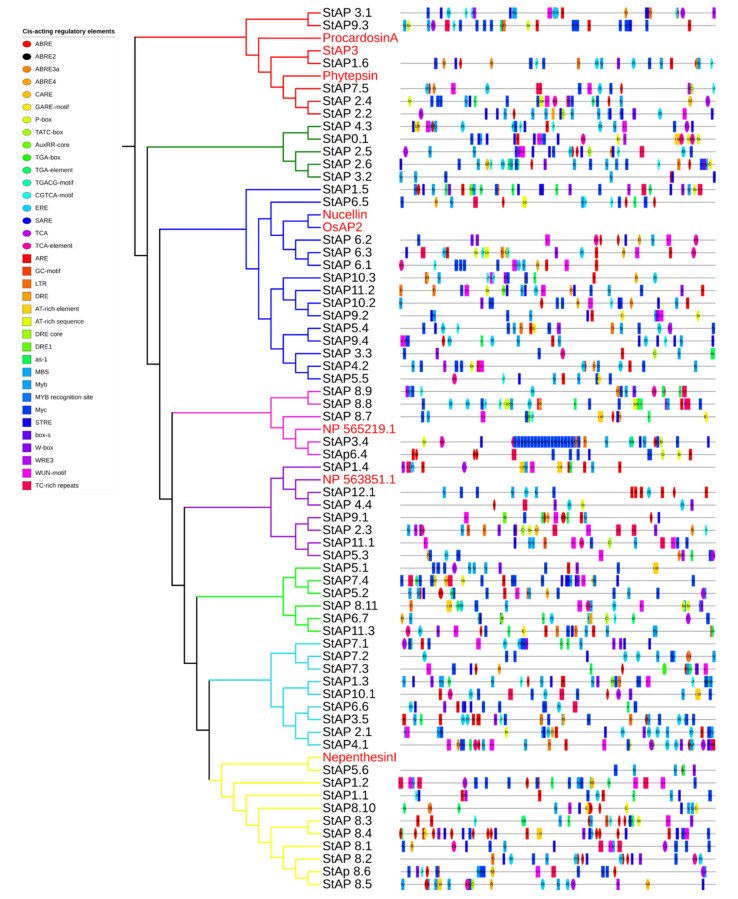
In silico identification of cis-acting elements in promoters of *St*AP. Colored boxes represent each element position in the coding strand.

Cis-acting elements were identified in silico in promoters of *S. tuberosum* aspartic proteases (Figure 5, Table S8), several of which correspond to abiotic and biotic stress responsiveness: anaerobic response element (ARE), enhancer element involved in specific anoxic inducibility (GC-motif), low-temperature response element (LTR), “cis-acting element involved in dehydration, low-temp, salt stresses” (DRE), water response elements (Myb, AT-rich element), drought response elements (DRE core, Dre1, as-1, MyC), MYB binding site involved in drought inducibility (MBS) and its drought stress-related MYB recognition site, “heat, osmotic stress, low pH, nutrient starvation stresses response element” (STRE), repetitions rich in TC involved in defense and response to stress (TC-rich repeats), and wound and pathogen response elements (WUN-motif, WRE3, W-box, Box S). In addition to stress-related elements, aspartic proteases presented motifs associated with phytohormone response: elements sensitive to abscisic acid (ABRE, ABRE2, ABRE3a, ABRE4), ABA and GA response element (CARE), cis-acting elements involved gibberellin response (GARE motif, P-box, and TATC box), auxin-responsive elements (AuxRR-core, TGA-box, TGA element), cis-acting regulatory element involved in the MeJA response (CGTCA-motif), ethylene-responsive element (ERE), and cis-acting elements involved in the response to salicylic acid (SARE, TCA, TCA element).

The majority of *St*AP coding genes presented more than one stress-related and phytohormonal-responsive cis-element in their promoter regions, some of them in very high copy numbers. In general, their presence correlates with the *St*APs expression profiles observed in Figure 4.

*St*APs that expressed differentially under NaCl and mannitol treatments (such as *St*AP1.6, *St*AP11.1, *St*AP5.3, and *St*AP8.11, among others) presented several cis-acting elements related to water, drought, salt, and osmotic stresses (AT-rich element, AT-rich sequence, DRE, DRE1, as-1, MBS, Myb, MYB recognition site, MyC, STRE). In addition, *St*AP1.6, *St*AP8.11, and *St*AP11.1 were also induced by ABA and presented cis-acting elements sensitive to abscisic acid, some of them in multiple copies (*St*AP1.6 has two copies of ABRE in the positive strand and three copies of ABRE, two of ABRE2, and one ABRE3a element in the negative strand; *St*AP8.11 has one ABRE4 in the positive strand and ABRE and ABRE3a in the negative strand, and *St*AP11.1 has one ABRE element in the negative strand). *St*AP1.6, which presented the highest copy number of abscisic acid-responsive elements, exhibited major induction. It has been described that more than one copy of ABRE is necessary for ABA-dependent gene expression [94].

Regarding heat stress response, promoters of *St*APs induced by heat (*St*AP7.5, *St*AP2.6, *St*AP2.1, and *St*AP8.7) presented one or more copies of the heat, osmotic stress, low pH, and nutrient starvation stresses response element (STRE). *P. infestans*-responsive *St*APs presented one, or in some cases two, cis-acting elements involved in defense, wound and pathogen responses (Box S, W box, WRE3, WUN-motif, TC-rich repeats). Salicylic acid-responsive elements (SARE, TCA, TCA-element) were present in three out of six upregulated and in five out of six downregulated genes under BTH treatments.

The presence of cis-acting regulatory elements found in *St*AP promoter regions is in agreement with previously reported roles of APs in water deficit, osmotic stress, and ABA signaling [45,85].

## 3. Materials and Methods

### 3.1. AP Identification and Characterization

A profile of Hidden Markov Model (HMMER 3.1b2, [95]) was created based on 59 aspartic protease amino acid sequences from *A. thaliana* [10]. These sequences were aligned with Multiple Alignment using Fast Fourier Transform (MAFFT) [96], and five of them were eliminated in order to improve the profile. The resulting alignment was trimmed on both ends, removing regions where the alignment was poor due to the variable nature. The HMMER profile was validated on the Arabidopsis TAIR database [97] (TAIR10_pep_20101214/) in accordance with sequences described [10,77].

Using this profile, an HMMER search was performed [98] against the Potato Genome Sequence Consortium reference genome Double monoploid DM1-3516-R44 [50] peptide database (Version 3.4). The HMMER logo was obtained with Skylign [99].

*St*APs were named according to their chromosome localization with a first number that indicates the chromosome and a second number that accounts for its relative position within the chromosome (i.e., the aspartic protease localized on the most upstream position of chromosome 2 is named *St*AP2.1).

### 3.2. Phylogenetic Analyses

Phylogenetic analyses were carried out with *St*AP amino acid sequences identified from *S. tuberosum* (PGSC DM peptide database, Version 3.4), and 8 reference AP plant amino acid sequences: Nucellin (U87148) [57], Procardosin A (CAB40134) [56], *St*AP3 (AAT77954) [11], *Os*AP2 (AAK81699) [27], Nepenthesin I (AB114914) [59], Phytepsin (CAA39602) [55], NP_563851.1 [23], and NP_565219.1 [10]. A second phylogenetic analysis included these sequences and *At*AP amino acids sequences (2005) [10].

Alignments and phylogenetic trees were performed with MAFFT [96]. A phylogenetic tree was generated using Simple Phylogeny with the neighbor-joining clustering method with 100 bootstrap replications and default parameters [100].

### 3.3. Domain Structure Analysis, MEME Motifs, and Subcellular Localization

AP protein domains and active sites were analyzed in Pfam database [101] and Scanprosite tool [102]. The Multiple EM for Motif Elicitation (MEME) online tool v5.2.0 [103] was employed to identify conserved motifs of *St*AP proteins. Parameter settings were as follows: optimum motif width was set to 6–50, the maximum number of motifs was set to 20, with expectation motif site distribution set to any number of repetitions and all other parameters set by default. SignalP V4.1 was used for identifying putative signal peptides [104]. PredoTar V1.04 [105], TargetP4.0 [106], ProtComp 9.0 [107] and YLOC [108,109] with default settings were used to predict signal sequences to organelles and other subcellular localizations [110].

### 3.4. Gene Structure Analysis

AP genes were checked for intron and exon structure based on primary transcripts from the PGSC [56].

### 3.5. Chromosome Localization and Duplication Analyses

A physical map was constructed with potato *St*APs gene localization obtained from the potato sequenced genome PGSC database (http://potato.plantbiology.msu.edu/data/PGSC_DM_V403_genes.gff.zip accessed date: 15 October 2020 [74]. Plots were generated with a custom gene viewer based on HTML5 Canvas, JQuery, and Kinetics. Js developed by the Agrobiotechnology Laboratory from INTA EEA-Balcarce.

For duplication analyses, genome tandem duplications were defined for those *St*AP genes falling within 50 Kb of one another [53]. While duplicated blocks were downloaded from the Plant Genome Duplication Database (PGDD) [80], the number of Ka and Ks values of non-synonymous and synonymous nucleotide substitutions, respectively, were extracted from PGDD, and Ka/Ks ratio was calculated. The duplication time was estimated according to the formula: T = Ks/2λ, assuming a clock-like rate (λ) was 6.9 × 10 ^−9^ substitutions/site/year for *S. tuberosum* [111].

### 3.6. Expression Profile

RNA-Seq expression data from double monoploid *S. tuberosum* group Phureja DM1-3 516 R44 clone [50,74] were integrated to local software (AgroBiotechTools from Agrobiotechnology Laboratory, INTA UIB-Balcarce) and represented as described in the graphical display. The data retrieved include libraries from a) abiotic stress conditions (salt, manitol and temperature); b) biotic stress (detached leaves inoculated with *P. infestans* (*Pi*), DL-β-aminobutyric acid (BABA), or benzoic-(1,2,3)-thiadiazol-7-carbotioic acid S-methyl ester (BTH) at 0 hs (biotic control) or a pool of samples at 24, 48, and 72 h post-inoculation (hpi) (biotic treatment)); c) hormonal treatments (indole acetic acid (IAA), gibberellic acid (GA3), 6-Benzylaminopurine (BAP), abscisic acid (ABA), or water (control)); and d) different tissues and plant organs (root, stem, leaf, stolon, primary and secondary tuber, flower, petiole, petal, carpel, sepal, callus, immature and mature fruits, and mesocarp and endocarp). Expression data were expressed in “Number of fragments per kilobase of exon per million fragments mapped” or FPKM [50,74].

### 3.7. Analysis of Cis-Acting Elements

Cis-acting elements were detected in the promoters of *St*AP by in silico analysis using the web software PlantCARE [112,113]. Upstream sequences of AP genes, -3000 bp to +103 bp from the start codon, were retrieved from the PGSC v4.03 database [74].

### 3.8. Graphical Displays

Protein domain structures, MEME motifs, expression analysis, cis-acting elements, and phylogenetic trees were plotted with iTOL program [81,82].

## 4. Conclusions

In the present study, we presented the first identification and description of 62 aspartic protease genes on the potato genome. The data obtained provide new knowledge about gene structure, chromosome localization, expression, and the evolution of the aspartic protease family in *S. tuberosum*, as well as *S. tuberosum* aspartic protease (*St*AP) protein organization, phylogenetic relations, and tissue and subcellular predicted localization.

Phylogenetic analysis revealed that *St*APs are distributed in eight groups, named from *St*API to *St*APVIII, that were differentiated into typical (*St*API), nucellin-like (*St*APIIIa), and atypical aspartic proteases (*St*APII, *St*APIIIb to *St*APVIII). Specific and common domains and Multiple EM for Motif Elicitation (MEME) motifs were defined among these groups.

Most *St*APs were predicted to be multi-located, most localized on the vacuole (29 *St*APs), plasma membrane (27 *St*APs), extracellular space (32 *St*APs), Golgi apparatus (21 *St*APs), and cytoplasm (14 *St*APs), while a few were in the nucleus (9 *St*APs), chloroplast (3 *St*APs), peroxisome (2 *St*APs), and mitochondria (1 *St*APs).

*St*APs were distributed along all 12 potato chromosomes. Segmental and tandem duplications evidenced in the present work, might have contributed to the expansion of the potato AP gene family.

A total of 27 intronless *St*AP genes were found, representing almost 44% of the total *St*AP mainly on atypical AP.

The RNAseq analysis revealed that 26 out of 62 genes presented a constitutive expression in different tissues or organs. Some *St*APs revealed preferential expression and tissue-specific patterns. Approximately 21% of *St*APs modified their gene expression profile under salt, osmotic, or temperature treatments. Gene expression was consistent with the presence of cis-acting regulatory elements on *St*AP promoter regions.

This study leads to a better understanding of the *St*AP family and provides the baseline material for future studies tending to determine the functional assignment of the identified genes, as well as the redundancy or diversification of functions in duplicate and tandem genes. Additionally, the knowledge of the diversity of *St*AP genes and their potential functions can be a source of information for future researchers whose objective is potato breeding through new biotechnology techniques such as gene editing mediated by CRISPR.

## Data Availability

All data generated or analyzed during this study are included in this published article [and its supplementary information files] or are available from the corresponding author on reasonable request.

## Supplementary Materials

The following are available online at https://www.mdpi.com/article/10.3390/plants11040544/s1, Table S1: Partial amino acid sequence alignment of *Solanum tuberosum* APs and *Arabidopsis thaliana* APs (with known function) and six references APs, Table S2: Segmental duplications, Table S3: Hidden Markov Model (HMMER3.1) profile tested against TAIR 10 2010-12-2014 peptide database, Table S4: Hidden Markov Model (HMMER3.1) profile tested against Potato Genome Sequence Consortium Double Monoploide 3.4 version (PGSC DM3.4) peptide database, Table S5: *St*AP list of selected proteins, Table S6: MEME motifs Logo and sequences, Table S7: Celullar and subcellular predicted localization of *St*AP based on YLoc plus Plants model, Table S8: Number of cis-acting regulatory elements present in *St*AP promoters.

## Abbreviations

AP: aspartic protease; PGSC: potato genome sequence consortium, MEME: Multiple EM for Motif Elicitation.
