# Supplementary material for: Genome-Wide Analyses of Aspartic Proteases on Potato Genome (Solanum tuberosum): Generating New Tools to Improve the Resistance of Plants to Abiotic Stress
Source: Plants (Basel). 2022 Feb 18;11(4):544. doi: 10.3390/plants11040544 (PMC8875628; doi:10.3390/plants11040544)
Supplement: Supplementary file 1 [file plants-11-00544-s001.zip › Table S1_final.pdf]

Table S1. Partial amino acid sequence alignment of Solanum tuberosum APs and Arabidopsis thaliana APs (with known function) and six reference APs. Sequences surrounding the first and second catalytic aspartate and the C-terminal side of the protease domain from A) Typical aspartic proteases; B) Nucelline-like; C) atypical aspartic proteases. The alignment was done using mafft [1] with default parameters with 100 maxiteration and it was visualized in Bioedit [2].

(a) Typical aspartic proteases

Protein ID	FIRST CATALYTIC ASPARTATE	SECOND CATALYTIC ASPARTATE	C-TERMINAL END
StAP3.1	IVYVKNYMDVQYFAEIGIGSPPQHFAV-<br>VFDTASSNLWVPSSKC	GCPAIVDTGTSFLAG-<br>PTTILTQI	PLWVLGDVFLRAYHTVFDFGN---<br>LQVGFAESA---
StAP9.3	IVYIKNYQDVQYFAE-<br>ISIGSPPQHFTVVFDTGSSNLWVPSSKC	GCPAIVDTGTAFIS-<br>GPTTILTQI	PLW------------------------<br>----------
Pro-CardosinA	VVALTNDRDTSYFGEI-<br>GIGTPPQKFTVIFDTGSSVLWVPSSKC	GCQAFADSGTSLLSGP<br>TAIVTQI	PLWILGDVFMRPYHTVFDYGN---<br>LLVGFAEAA---
StAP7.5	IIALKNYLDAQYFGEISIGSPPQKFTVIFD<br>TGSSNLWVPSAKC	GCSAIADSGTSLLAG-<br>PTTIITQI	PLWILGDVFMGRYHTVFDSEK---<br>MRVGFAEAA---
Phytepsin	IVALK-<br>NYMNAQYFGEIGVGTPPQKFTVIFDTGSS<br>NLWVPSAKC	GCAAIADSGTSLLAG-<br>PTAIITEI	PLWILGDVFMGPYHTVFDYGK---<br>LRIGFAKAA---
StAP2.4	IVALK-<br>NYMDAQYFGEIGVGTPPQKFTVIFDTGSS<br>NLWVPSAKC	GCSAIADSGTSLLAG-<br>PTTVITMI	PLWILGDVFMGRYHTVFDSGK---<br>LRVGFAEAA---
StAP2.2	IVALK-<br>NYMDAQYFGEIGVGTPPQKFTVIFDTGSS<br>NLWVPSSKC	GCSAIADSGTSLLAG-<br>PTTVITMI	PLWILGDIFMGRYHTVFDYGK---<br>LRVGFAEAA---
StAP1.6	IVPLKNYLDAQYYGEIGIGSPPQKFKII-<br>FDTGSSNLWVPSSKC	GCAAIVDSGTSLLAG-<br>PTTIVTQI	PLWILGDVFMGPYHTVFDYGK---<br>SQVGFAEAA---
StAP3	IVPLKNYLDAQYYGEI-<br>GIGSPPQKFKVIFDTGSSNLWVPSSKC	GCAAIVDSGTSLLAG-<br>PTTIVAQI	PLWILGDVFMGPYHTVFDYGK---<br>SQVGFAEVHNSL

(b) Nucelline-like aspartic proteases.

Protein ID	FIRST CATALYTIC ASPARTATE	SECOND CATALYTIC ASPARTATE	C-TERMINAL END
StAP6.5	GNIHSEGLYYTYMLVGNPPR--<br>PYFLDIDTGSDLMWIQCDAP	VFDSGSTY-<br>TYFTDQAY	ILGDISLRGQLFVYDNVNQKIGWIRSNCE------<br>R
NP_564539.1_APCB1	GNVYPDGLYYTRILVGK-<br>PEDGQYYHLDIDTGSELTWIQCDAP	LFDTGSSYT-<br>YFPNQAY	ILGDISMRGHLIVYDNVKRRIGWMKSDCV-----
StAP6.3	GNVYPIGYYQVTLEVGQPPK--PYFFD-<br>IDTGSDLTWLQCDAP	VFDSGSTFT-<br>YFSSKAY	LIGDISLLDKMVVYDNEKKQIGWLPANCK------<br>R
StAP6.1	GNVYPKGYYQVTLNVGQPPK--<br>PYFLDIDTGSDLTWLQCDAP	ILDSGSTFT-<br>YFNSKAY	LIGDISMLDKLVIYDNEKQQIGWAPANCN------<br>R
StAP6.2	GNVYPLGYYYVQLNIGQPSR--<br>PFFLDPDTGSDLTWLQCDAP	VFDSGSS-<br>FSYLNAHTY	LIGDISMQDKMVIYDNEKQAIGWTSANCD------<br>R
Nucellin	GNVYPVGHFYATLNIGEPAK--<br>PYFLDVDTGSNLTWLECHHP	VFDSGSTY-<br>THVPAQIY	LIGAVTMQDLFVIYDNEKKQLGWVRAQCD------<br>R
OsAP2	GNVYPIGHFFVTMNISDPAK--<br>PYFLDIDTGSTLTWLQCDYP	IFDSGATY-<br>TYFALQPY	LIGGITMLDQMVIYDSERSLLGWVNYQCD------<br>R
StAP1.5	DDLLINGYYTTHIWIGTPPQ--<br>KFALIVDTGSTVTYVPCSS-	ILDSGTTYAY<br>LPEAVF	LLGGIVVRNTLVTYDRENERIGFWKTNCS-<br>ELWDR

(c) Atypical aspartic proteases

Protein ID	FIRST CATALYTIC ASPARTATE	SECOND CATALYTIC ASPARTATE	C-TERMINAL END
StAP1.3	EYFTRIGVGTPPK-<br>YVYMVLDTGSDVVWIQCSP	-AGDGGVIVDSGTSVTRL-<br>TRPAYVALRDSFR	SGLSIIGNIQQQGFRVVFDLAGSRLG-<br>FAPRGCA--

StAP10.1	EYFTRIGIGTPTK–YVYL-<br>VLDTGSDIVWIQCLP	-EGNGGVIVDSGTSVTRL-<br>TRPGYIAVRDAFR	GGLSIIGNIQQQGFRVVFDLAGSRLG-<br>FAPRGCSY-
StAP6.6	EYFARLGVGEPAK–<br>EFYMAIDTGSDINWLQCEP	-NGRGGIIVDSGTAVTRLQS-<br>SAYNALRDTFV	ESMSIIGNVQQQGTRVSYDLT-<br>NNLVGFSLDKC---
StAP3.5	EYFTRLGLGQPVK–<br>EYYMVLDTGSDITWVQCEP	-SGSGGIIVDSG-<br>TAVTRLKSTAYNTLRDSFV	GSLSIIGNIQQQGTRVSYDLANNLVGF-<br>SPDKC---
StAP2.1	EYFVRIGVGSPVR–<br>QQYMVIDAGSDIVWVQCQP	-EGDGGVVMDTGTAVTRL-<br>PQEAYVAFRDAFA	TRLSIIGNIQQEGIQI-<br>SIDGANGFVGFGPNIC---
StAP4.1	EYFVRIGVGSPAK–<br>NQYMVLDSGSDIVWVQCQP	-LGDGGVVMDTG-<br>TAVTRLPSEAYVAFRDAFI	SKLSIIGNIQQEGI-<br>QISFDGANGFVGFGPNIC---
StAP5.6	EFLMQISIGSPSE–SYNAIM-<br>DTGSDLIWTQCKP	-DGTGGMI-<br>IDSGTTITYLEESAFSLLKKEF<br>S	SGMSIFGNVQQQNMMVI-<br>HDLDKETLSFVPTQCDKL
Ne-pen-thesinI	EYLMNLSIGTPAQ–PFSAIM-<br>DTGSDLIWTQCQP	-NGTGGIIIDSGTTLTY-<br>FVNNAYQSVRQEFI	QGMSIFGNIQQQNMLVVYDTGNSVVSFAS<br>AQCGAS
StAP7.4	EYFMDVFVGTPPK–<br>HFSLILDTGSDLNWLQCVP	-EGVGGTIIDSGTTLSY-<br>FAEPAYEIIKEAFV	SALSIIGNYQQQNFHILYDTKRS-<br>RLGYAPTRCADA
StAP5.2	EYFMDVFVGTPPK–<br>HFSLILDTGSDLNWIQCVP	-QGVGGTIIDSGTTLSY-<br>FIEPAYEIIKEAFV	SAMSIIGNYQQQNFHILYDTKRS-<br>RLGYAPTKCADA
StAP1.1	EYLMRYSIGTPPV–<br>HTLGVADTGSDLTWTQCLP	--GEGNIIIDSGTVLTLF-<br>STEIYVKLESMVK	YGGQIFGNIAQMNFLVGYDLK-<br>NKRVSFKATDCSK-
NP_198319	EYLMNVSIGTPPF–<br>PIMAIADTGSDLLWTQCAP	--SEGNIIIDSGTTLT-<br>LLPTEFYSELEDAVA	PSFSIYGNVAQ-<br>MNFLVGYDTVSKTVSFKPTDCAKM
StAP8.10	DYLMKIAIGTPPR–<br>KTYAILDTGSELTWIQCKP	DEDLGNIIIDSGTTLTFL-<br>PENFYNKFESILV	GQYGVYGNLAQMN-<br>FLIGYDLVNRKLSFLPSDCTKH
StAP8.3	EYLMKLSIGTPPV–<br>EIVAIADTGSDLTWTQCKP	GGEEGNIIIDSGTTLT-<br>FLPNDFYSSLESTLV	--------------------------------<br>-------
StAP8.4	EYLMKLSIGTPPV–<br>EIVAIADTGSDLTWTQCMP	DGQAGNIIIDSGTTLTLL-<br>PNDFYSNLESTLV	DEIAIFGNLAQGNFLIEYDL-<br>VANKISFQPTDCTKY
StAP8.1	---MKISIGTPPI–<br>DIFAFADTGSDLTWTQCEP	--PQGNIIIDSGTTLT-<br>LVPVDFYAKLEKTLV	EEVSILGNLAQMNLLIGFDL-<br>VANKVSFLPTDCTKH
StAP8.6	EFLMKISIGTPPI–DTFVI-<br>ADTGSDLTWTQCKP	GYDRGNIIIDSGTTLTYVPDVF<br>YLNLESLLI	NQISILGNLAQANFLIGFDLEANK-<br>MSFKPTDCTKY
StAP8.5	EFLMKISIGTPPI–DTFVI-<br>ADTGSDLIWTQCEP	GDDLGNI-<br>VIDSGTTLTYVPDGFYLN-<br>LESLLM	NQISILGNLAQAN-<br>FLIGYDLKAYKVSFKPTDCTKY
StAP8.2	EYLMKISIGTPPI–ETLVI-<br>ADTGSDLTWTQCKP	GYDRGNIIIDSGTTLTYVPDGF<br>YLNLESLLM	NQISILGNLAQANFLIGYDL-<br>KANRVSFKRTDCTKY
StAP1.2	VYLTKISIGTKPS–<br>AKLVAVDTGSDLIWIQCEP	-HKGGKIAIDSGTTFTFLPTH-<br>MYSEMETIMK	KHLPIFGNIAQTNFFVAYDLD-<br>NKTVSFKPQICAKL
StAP3.4	NYIVTVTLG--GR–<br>NMTVIVDTGSDLTWVQCQP	--KSGFLIDSGTVITRL-<br>PPSIYKAVKAEFL	GEIGIIGNFQQRNTRVIYDTKQSQVG-<br>FAKETCSFM

StAP6.4	NYIVTVTLG--GR–NTTVIVDTGSDLTWVQCQP	--GKSGMIIDSGTVITRL-PPTIYKAVKAEFL	DEIGIIGNYQQKNTRVIYDIKQSQVG-FAKETCSF-
NP_565219	NYIVTVELG--GK–NMSLIV-DTGSDLTWVQCQP	--GR-GILIDSGTVITRL-PPSIYKAVKIEFL	NEVGIIGNYQQKNQRVI-YDTTQERLGIVGENCRV-
StAP8.7	NYIVTIGIGTPKK–DLT-LIFDTGSDLTWTQCEP	--KNAG-TIIDSGTVITRLPSTAYANMRATFR	DSIGIFGN-TQQQTMEIVYDVAGEKLGFGYGGCT--
StAP5.1	QYFVDLRLGTPPQ–RLLLVADTGSDLVWVSCSA	-LGNGGTVMDSGTTLT-FLAEPAYRRIVQAFK	SGFSVIGNLMQQGFMFEFDRDQSRIGFSRHGCG--
StAP12.1	NYVVRVKMGTPGQ–QMYMVLDTSNDAAFVPCGG	-NTGAG-TVIDSGTVITRFVQPAYEAIRDEFR	SVLNVIANLQQQNLRIM-FDTANSRVGIAHEVCN--
StAP4.4	NYVVRAKIGTPGQ--LMFMVLDTSSDTAWVPCSG	-NTGAGTIIDSGTVITRFVE-PIYNAIRDEFR	SVLNVIANLQQQNLRILFDTANSRLGI-ARELCN--
NP_563851AED3	NYVVRAKLGTPPQ--LMFMVLDTSNDAVWLPCSG	-NSGAGTIIDSGTVITRFA-QPVYEAIRDEFR	AVLNVIANLQQQNL-RILFDVPNSRIGIAPEPCN--
StAP1.4	TYIVKAKIGTPPQ--TLLVALD-NSNDFSWFPCGG	-NTGAGTIIDSGTVFTRLVE-PAYTAVRNEFR	SVLNVI-ASWQQQNHRILFDVPNSRVGVARETCS--
NP_187876NANA	QYFTEIRVGTPAK--KFRVVVDTGSELTWVNCRY	-TSGGGTILDSGTSLT-LLADAAYKQVVTGLA	PATNVIGNIMQQNYLWEFDL-MASTLSFAPSACT--
StAP6.7	GYSIALSFGTPPQ--KIPFIM-DTGSNFVWFPCTT	-NGNGGSIVDSGT-FTFMNRGVFEPVLDAFV	GPSIILGNFQMQNYLVEFDLK-NEKFGFKQQMCK--
StAP11.3	GYSITLPFGTPPQ---MPFVMDTGSSFVWFPCTK	-KGNGGTIVDSGT-FTFLNHDIFVPVMNAFV	GPSIILGNFQMQNFFVEFDLKNNMFG-FRDQVCK--
StAP8.11	DYTLSFSLGSQ----TISLYMDTGSDVVWLPCHP	-RGNGGMVVDSGTTFT-MLPLKFYETVVTEFV	GPAGILGNYFQQGFEVVYDLEKKRVG-FAKRKCA--
StAP9.1	TLTVSLSVGTPPQ--NVTMVLDTGSELSWLHSN-	-TGAGQTMVDSGTQFTFLLGPAYTALRSEFL	IEAYIIGHHHQQNVWVEYDLEKSRIG-FAQVQCNIA(8)
NP195839PCSC1	TLTVTLTVGTPPQ--NISMVIDTGSELSWLRCN-	-TGAGQTMVDSGTQFTFLLGPVYTALRSHFL	MEAYVIGHHHQQNMWIEFDLQRS-RIGLAPVECDVS(8)
StAP2.3	SLTVSLSIGSPPQ--QVVMVLDTGSELSWLHCK-	-TGAGQTMVDSGTQFTYLLGPAYTALKNEFI	VAAYII-GHHHQQNFWMEFDLANSRVGLAQVS-CELA(8)
StAP11.1	ALIVTLPIGTPPQ--DQQMVLDTGSQLSWIQCN-	-GGSGQTMIDSGTQYTFLVEEAYSKVRDEVV	IASNIIGNFHQQNLWVEFDLS-NKRVGFGKVECK---
StAP5.3	------------------MVLDTGSQLSWIQCH-	-GGSGQTIVDSGTEY-TFLVEEAYNKVREEIV	IASNIIGNFHQQNLWVEFDLRN-RRVGFGKGECSMQV
StAP7.1	EYVASFLIGTQMI--KNYLLLDTGSDLIWWQCGP	-MMNGGMFVDTGTS	SERSVLGMTQLQGVGLTFDTISSTLSFD-IDACD--

StAP7.2	EYIAT-FNLGTDDHKFKRYLLVDTGSDLVWWQCGP	-AGDGGVIVDSGTSVTRL-TRPAYVALRDSFR	NPVTIIGSYQLQGVGLTFDTATDTLSFD-IDACGAD
StAP7.3	EYVATFTIGAEET--KSFLLIDTGSDLVWWQCKP	-EGNGGVIVDSGTSVTRL-TRPGYIAVRDAFR	QNVAIIGNHQLQGVGLTFDTGTD-TLSFDLDAC---
StAP8.9	GYTVKIGLGTPRQ--NFFLMFDTGSPATWVRCKS	-IGNGGVIIDSGTSVTRLIR-PAYIAMRDAFR	DNLSIIGSVQQRGMNVIYDLKQKRIGFG-TNCPTY
StAP8.8	LFTVKISLGSPQQ--YYSLIV-DTGSRWTWVRCRF	-TGNGGVIIDSGTSVTRL-TQPAYVALRDAFR	ESYSIIGIVQQRGFNVLYDLEWKTIGFG-TNCT--
StAP11.2	LYFTRVQLGSPPK--EFYVQIDTGSDVLWVSCAS	-NGRGGIIVDSGTAVTRLQS-SAYNALRDTFV	QGLTILGDLVLKDKIIVYDLAGQRIG-WANYDCSQA(53)
StAP10.2	FYYTKVKLGSPPR--EYNVQIDTGSDILWVTCGS	-SGSGGVILDCG-TAVTRLQTQAYNSLRDAFL	LGLTILGDLVLKDKIIVYDLARQRIG-WAEYDCSSP(65)
StAP9.2	LYFTKVRLGTPPR--EYNVQIDTGSDILWVTCSS	-SGSGGIIIDSG-TAVTRLQTEIYNSLRDSFV	QGVTILGDLVLKDKIIVYDLARQRIG-WTDYDCSSS(53)
StAP10.3	IYYTKVKVGSPPR--EYNLLIDTGSEISWIACSS	-LGDGGVVMDTG-TAVTRLPSEAYVAFRDAFI	PSLTVLGDIALRDKSIVYDLAHQRIG-WADHDCSLP(34)
StAP3.3	LYFTKLSIGTPSK--DY-HVQVDTGSDLLWLNCAG	-DGTGGMI-IDSGTTITYLEETAFKVLKEEFT	DELYLLGDLVLSNKLVLYDLEKKTL-GWTQYDCSSN(42)
StAP4.2	LYYTKLTIGTPSK--DY-HVQVDTGSDILWVNCAG	-NGTGGIIIDSGTTLTY-FVNNAYQSVRQEFI	KEITLLGDLVLSNKLVLYDLENQSIG-WTEYNYI--
StAP5.5	LYYTKISIGTPSR--DFHVQVDTGSDILWVNCAS	-EGVGGTIIDSGTTLSY-FAEPAYEIIKEAFV	SEITLLGDLALSNKLVVYDIENQTIG-WTEYDCSST(47)
StAP5.4	LYFAEIGIGTPPN--NYFVQVDTGSDIMWVNCIA	--GEGNIIIDSGTVLTLF-STEIYVKLESMVK	WNLIVFGDLVLSNRLILYDLENQAIG-WTEYNCSSN(46)
StAP9.4	LYYAKIGIGTPSN--GYYVQVDTGSDIMWVNCVG	--GEGNIVIDSGTTLT-LLPSNFYYELESVVA	WNLTVFGDLVLSNKLVLYDLEKQAIG-WTEYNCSSS(46)
StAP2.6	LHYANVTVGSPGL--SFLVALDTGSDLFWLPCD-	DEDLGNIIIDSGTTLTFL-PENFYNKFESILV	--VNIIGQNFMTGYRVIFDREKMVL-GWKASDCYDS(84)
StAP3.2	LHYANVTVGTPGL--SFLVALDTGSDLFWLPCN-	GGEEGNIIIDSGTTLT-FLPNDFYSSLESTLV	--VNIIGQNFMTGYQVVFDREKMVL-GWKPSDCYDS(80)
StAP2.5	LHYTTVTLGTPGL--KFLVALDTGSDLFWVPCE-	GDDLGNI-VIDSGTTLTYVPDGFYLN-LESLLM	--LNIIGQNFMTGYRFVFDREKIIL-GWKKFDCYDY(69)
StAP4.3	LHYMWVDIGTPNV--SFLVAL-DAGSDLLWVPCE-	SKRTGNIIIDSGTTLT-LLDSGFYDDFGTAVE	-EIGTIGQNFMTGYRMVFDRENLKL-GWSRSDCEDL(81)
StAP0.1	LHYVWIDIGTPNA--SFLVAL-DAGSDLLWLPCN-	--LEGNIVIDSGTTLTY-FPVSYCNLVRQAVE	------------------------------------
