# Supplementary material for: Genome-Wide Analyses of Aspartic Proteases on Potato Genome (Solanum tuberosum): Generating New Tools to Improve the Resistance of Plants to Abiotic Stress
Source: Plants (Basel). 2022 Feb 18;11(4):544. doi: 10.3390/plants11040544 (PMC8875628; doi:10.3390/plants11040544)
Supplement: Supplementary file 1 [file plants-11-00544-s001.zip › Table S2_final.pdf]

Table S2. Segmental duplications. Data obtained from PGDD (http://chibba.agtec.uga.edu/duplication/index/downloads) Ka/Ks was calculated and duplication date was estimated as was explained in Materials and Methods.

Block_N°	Block Score	E_value	Locus_1	Locus_2	Ka	Ks	Ka/Ks	Approx. duplication date (MYA)
193	222.0	9,00E-167	*St*AP11.2	*St*AP9.2	0.31	1.47	0.21	−106.52
33	1213.0	3,00E-119	*St*AP2.5	*St*AP3.2	0.51	1.92	0.27	139.13
56	339.0	2,00E-146	*St*AP3.5	*nd	0.37	1.82	0.20	131.88
126	400.0	5,00E-173	*St*AP6.7	*St*AP11.3	0.18	0.75	0.24	54.35
30	506.0	0.0	*St*AP2.4	*St*AP2.2	0.08	0.51	0.16	36.96
62	644.0	0.0	*nd	*St*AP6.4	0.15	0.69	0.22	50.00
201	381.0	3,00E-137	*nd	*St*AP4.4	0.12	0.78	0.15	56.52

*nd were detected by HMM search but were eliminated from the *St*AP list.
Ka= non synonymous substitution rate.
Ks= synonymous substitution rate.
